# 10-Methyl-2-oxo-4-phenyl-2,11-di­hydro­pyrano[2,3-*a*]carbazole-3-carbo­nitrile

**DOI:** 10.1107/S1600536813011823

**Published:** 2013-05-04

**Authors:** A. Thiruvalluvar, E. Yamuna, R. Archana, K. J. Rajendra Prasad, R. J. Butcher

**Affiliations:** a Postgraduate Research Department of Physics, Rajah Serfoji Government College (Autonomous), Thanjavur 613 005, Tamilnadu, India; b Department of Chemistry, Tamkang University, Tamsui 25137, Taiwan; c Department of Chemistry, Bharathiar University, Coimbatore 641 046, Tamilnadu, India; d Department of Chemistry, Howard University, 525 College Street NW, Washington, DC 20059, USA

## Abstract

In the title mol­ecule, C_23_H_14_N_2_O_2_, the atoms in the carbazole unit deviate from planarity [maximum deviation from mean plane = 0.1018 (8) Å]. The pyrrole ring makes dihedral angles of 4.44 (5), 3.84 (5), 2.18 (5) and 56.44 (5)° with the pyran, fused benzene rings and phenyl ring, respectively. In the crystal, pairs of N—H⋯O hydrogen bonds generate *R*_2_^2^(14) loops and a C—H⋯N inter­action is also found. Mol­ecules are further linked by a number of π–π interactions [centroid–centroid distances vary from 3.5702 (5) to 3.7068 (6) Å], forming a three-dimensional network.

## Related literature

For a related structure, see: Sridharan *et al.* (2009 ▶). For hydrogen-bond motifs, see: Bernstein *et al.* (1995 ▶).
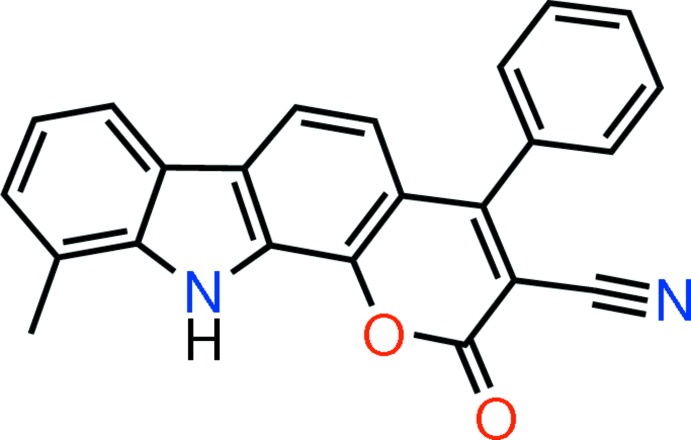


## Experimental

### 

#### Crystal data

C_23_H_14_N_2_O_2_
*M* *_r_* = 350.36
Monoclinic, 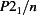

*a* = 7.8659 (1) Å
*b* = 8.5151 (1) Å
*c* = 25.1137 (4) Å
β = 98.133 (2)°
*V* = 1665.17 (4) Å^3^
*Z* = 4
Mo *K*α radiation
μ = 0.09 mm^−1^
*T* = 123 K
0.46 × 0.41 × 0.29 mm

#### Data collection

Agilent Xcalibur Ruby Gemini diffractometer
Absorption correction: multi-scan (*CrysAlis PRO*; Agilent, 2012 ▶) *T*_min_ = 0.978, *T*_max_ = 1.000
27185 measured reflections
8485 independent reflections
7184 reflections with *I* > 2σ(*I*)
*R*_int_ = 0.022

#### Refinement

*R*[*F* ^2^ > 2σ(*F* ^2^)] = 0.053
*wR*(*F* ^2^) = 0.154
*S* = 1.16
8485 reflections
249 parameters
H atoms treated by a mixture of independent and constrained refinement
Δρ_max_ = 0.64 e Å^−3^
Δρ_min_ = −0.31 e Å^−3^

### 

Data collection: *CrysAlis PRO* (Agilent, 2012 ▶); cell refinement: *CrysAlis PRO*; data reduction: *CrysAlis PRO*; program(s) used to solve structure: *SHELXS97* (Sheldrick, 2008 ▶); program(s) used to refine structure: *SHELXL2013* (Sheldrick, 2008 ▶); molecular graphics: *ORTEP-3 for Windows* (Farrugia, 2012 ▶) and *PLATON* (Spek, 2009 ▶); software used to prepare material for publication: *SHELXL2013* and *PLATON*.

## Supplementary Material

Crystal structure: contains datablock(s) global, I. DOI: 10.1107/S1600536813011823/sj5318sup1.cif

Structure factors: contains datablock(s) I. DOI: 10.1107/S1600536813011823/sj5318Isup2.hkl

Supplementary material file. DOI: 10.1107/S1600536813011823/sj5318Isup3.cdx

Supplementary material file. DOI: 10.1107/S1600536813011823/sj5318Isup4.cml

Additional supplementary materials:  crystallographic information; 3D view; checkCIF report

## Figures and Tables

**Table 1:** Hydrogen-bond geometry (Å, °)

*D*—H⋯*A*	*D*—H	H⋯*A*	*D*⋯*A*	*D*—H⋯*A*
N11—H11⋯O2^i^	0.874 (18)	2.095 (19)	2.9561 (11)	168.2 (15)
C43—H43⋯N31^ii^	0.95	2.56	3.3130 (17)	136

Symmetry codes: (i) 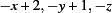; (ii) 
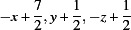
.

**Fig. 1.**
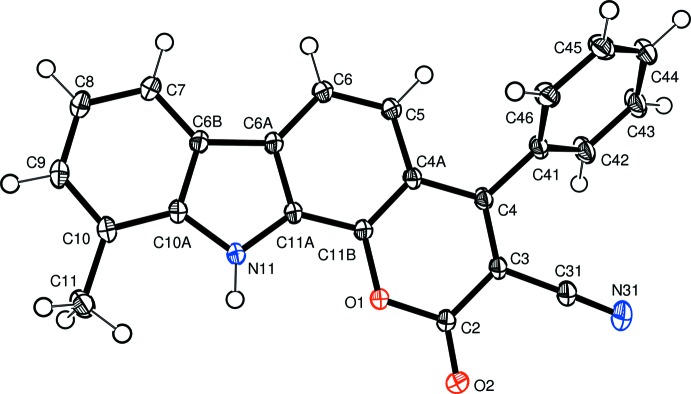
The molecular structure of the title compound, with displacement ellipsoids drawn at the 50% probability level. H atoms are shown as small spheres of arbitrary radius.

**Fig. 2.**
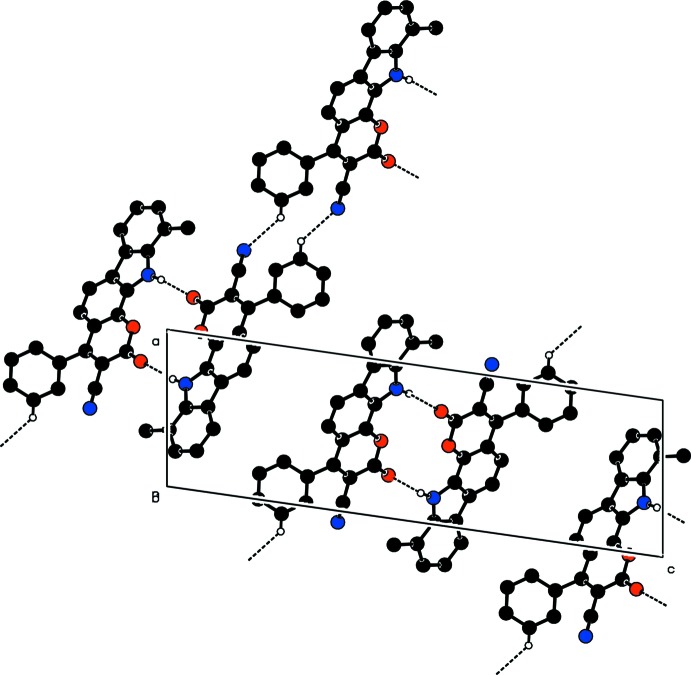
The partial packing of the title compound, viewed down the b axis. Dashed lines indicate hydrogen bonds. H atoms not involved in hydrogen bonding have been omitted.

**Fig. 3.**
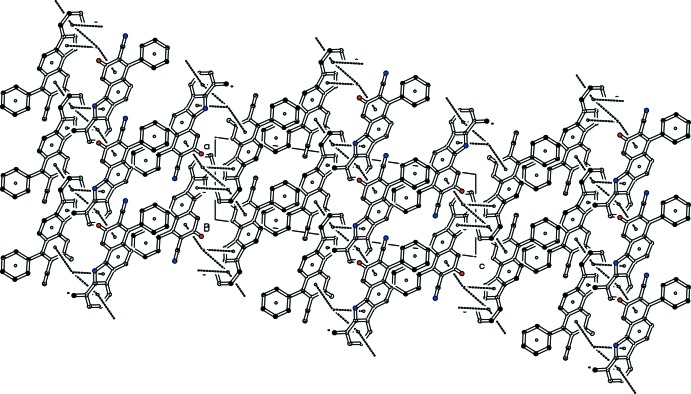
The crystal structure of compound, showing the formation of π-π stacking interactions.

## supplementary crystallographic information

### Comment

The title compound has been analysed as part of our crystallographic studies on pyranocarbazoles. Sridharan *et al.* (2009), have reported the synthesis and X-ray crystal structure of a related pyranocarbazole.

In the title molecule (Scheme I, Fig. 1), C_23_H_14_N_2_O_2_, the atoms in the carbazole unit deviate from planarity [maximum deviation from mean plane = -0.1018 (8) Å for atom C4A]. The pyrrole ring makes dihedral angles of 4.44 (5), 3.84 (5), 2.18 (5) and 56.44 (5)° with the pyran, fused benzene rings and phenyl ring, respectively.

Intermolecular N11—H11···O2 hydrogen bonds form a *R*^2^_2_(14) (Bernstein *et al.*, 1995) ring in the crystal structure and a C43—H43···N31 interaction is also found (Table 1, Fig. 2). Molecules are further linked by five π-π [*Cg*1—*Cg*4^i^ = 3.7068 (6), *Cg*2—*Cg*4^ii^ = *Cg*4—*Cg*2^iii^ = 3.5702 (5), *Cg*4—*Cg*1^i^ = 3.7067 (6) and *Cg*4—*Cg*4^i^ = 3.5927 (6) Å, symmetry code (i): 1 - *x*, 2 - *y*, - *z*, (ii): 1 + *x*, *y*, *z*, (iii): - 1 + *x*, *y*, *z* where *Cg*1, *Cg*2 and *Cg*4 are the centroids of the pyrrole (N11/C11A/C6A/C6B/C10A), pyran (O1/C2/C3/C4/C4A/C11B) and benzene (C6B/C7—C10/C10A) rings, respectively (Fig. 3)] interactions to form a three-dimensional network.

### Experimental

A mixture of benzaldehyde (0.106 g, 1 mmol), malononitrile (0.080 g, 1.2 mmol), 8-methyl-9*H*-carbazol-1-ol (0.197 g, 1 mmol) and NaHCO_3_ (0.084 g, 2 mmol) was ground at room temperature with the mortar and pestle. The reaction was monitored by TLC. After the completion of the reaction, the mixture was poured into water and then filtered. The obtained crude product was purified by silica gel column chromatography using petroleum ether: ethyl acetate (98:2) yielded the title compound (0.308 g, 88%). Then this pure compound was recrystallized from EtOAc.

### Refinement

The H atom bonded to N11 was located in a difference Fourier map and refined freely; N11—H11 = 0.874 (18) Å. Other H atoms were positioned geometrically and allowed to ride on their parent atoms, with C—H = 0.95–0.98 Å, and with *U*_iso_(H) = 1.2–1.5*U*_eq_(parent atom).

### Crystal data

**Table:**

C_23_H_14_N_2_O_2_	*F*(000) = 728
*M**_r_* = 350.36	*D*_x_ = 1.398 Mg m^−^^3^
Monoclinic, *P*2_1_/*n*	Melting point: 573 K
Hall symbol: -P 2yn	Mo *K*α radiation, λ = 0.71073 Å
*a* = 7.8659 (1) Å	Cell parameters from 11879 reflections
*b* = 8.5151 (1) Å	θ = 3.3–37.6°
*c* = 25.1137 (4) Å	µ = 0.09 mm^−^^1^
β = 98.133 (2)°	*T* = 123 K
*V* = 1665.17 (4) Å^3^	Prism, colourless
*Z* = 4	0.46 × 0.41 × 0.29 mm

### Data collection

**Table:**

Agilent Xcalibur Ruby Gemini diffractometer	8485 independent reflections
Radiation source: Enhance (Mo) X-ray Source	7184 reflections with *I* > 2σ(*I*)
Graphite monochromator	*R*_int_ = 0.022
Detector resolution: 10.5081 pixels mm^-1^	θ_max_ = 37.7°, θ_min_ = 3.3°
ω scans	*h* = −12→13
Absorption correction: multi-scan (*CrysAlis PRO*; Agilent, 2012)	*k* = −14→11
*T*_min_ = 0.978, *T*_max_ = 1.000	*l* = −33→42
27185 measured reflections	

### Refinement

**Table:**

Refinement on *F*^2^	Primary atom site location: structure-invariant direct methods
Least-squares matrix: full	Secondary atom site location: difference Fourier map
*R*[*F*^2^ > 2σ(*F*^2^)] = 0.053	Hydrogen site location: mixed
*wR*(*F*^2^) = 0.154	H atoms treated by a mixture of independent and constrained refinement
*S* = 1.16	*w* = 1/[σ^2^(*F*_o_^2^) + (0.0629*P*)^2^ + 0.6142*P*] where *P* = (*F*_o_^2^ + 2*F*_c_^2^)/3
8485 reflections	(Δ/σ)_max_ = 0.001
249 parameters	Δρ_max_ = 0.64 e Å^−^^3^
0 restraints	Δρ_min_ = −0.31 e Å^−^^3^

### Special details

**Table:**

Geometry. Bond distances, angles *etc*. have been calculated using the rounded fractional coordinates. All su's are estimated from the variances of the (full) variance-covariance matrix. The cell e.s.d.'s are taken into account in the estimation of distances, angles and torsion angles

### Fractional atomic coordinates and isotropic or equivalent isotropic displacement parameters (Å^2^)

**Table:**

	*x*	*y*	*z*	*U*_iso_*/*U*_eq_	
O1	1.01566 (8)	0.60784 (8)	0.06827 (3)	0.0167 (2)	
O2	1.22719 (10)	0.44963 (10)	0.05318 (3)	0.0242 (2)	
N11	0.66938 (9)	0.73028 (9)	0.03766 (3)	0.0157 (2)	
N31	1.57295 (15)	0.47611 (18)	0.15540 (5)	0.0423 (4)	
C2	1.17839 (11)	0.55044 (11)	0.08165 (4)	0.0171 (2)	
C3	1.27720 (11)	0.61006 (11)	0.13093 (4)	0.0172 (2)	
C4	1.21143 (10)	0.71613 (11)	0.16390 (4)	0.0155 (2)	
C4A	1.04339 (10)	0.78085 (10)	0.14642 (4)	0.0151 (2)	
C5	0.97078 (11)	0.90356 (11)	0.17439 (4)	0.0177 (2)	
C6	0.80984 (11)	0.96194 (11)	0.15617 (4)	0.0183 (2)	
C6A	0.71503 (10)	0.89666 (10)	0.10970 (4)	0.0156 (2)	
C6B	0.54287 (10)	0.92067 (10)	0.08278 (4)	0.0160 (2)	
C7	0.40849 (12)	1.01896 (12)	0.09342 (4)	0.0203 (2)	
C8	0.25297 (12)	1.00848 (13)	0.06019 (4)	0.0228 (2)	
C9	0.23100 (12)	0.90200 (12)	0.01695 (4)	0.0213 (2)	
C10	0.36192 (11)	0.80389 (11)	0.00460 (4)	0.0178 (2)	
C10A	0.51917 (10)	0.81560 (10)	0.03876 (4)	0.0155 (2)	
C11	0.33434 (14)	0.69327 (13)	−0.04214 (4)	0.0243 (2)	
C11A	0.78675 (10)	0.77783 (10)	0.08058 (4)	0.0145 (2)	
C11B	0.95174 (10)	0.72217 (10)	0.09862 (4)	0.0144 (2)	
C31	1.44170 (13)	0.53773 (14)	0.14491 (4)	0.0252 (3)	
C41	1.30970 (11)	0.76005 (11)	0.21667 (4)	0.0171 (2)	
C42	1.47693 (12)	0.81736 (14)	0.21961 (4)	0.0231 (2)	
C43	1.57064 (13)	0.85772 (15)	0.26898 (5)	0.0265 (3)	
C44	1.49880 (14)	0.83808 (14)	0.31591 (4)	0.0249 (3)	
C45	1.33329 (14)	0.77825 (13)	0.31351 (4)	0.0234 (2)	
C46	1.23742 (12)	0.74058 (12)	0.26415 (4)	0.0203 (2)	
H5	1.03437	0.94587	0.20613	0.0213*	
H6	0.76336	1.04525	0.17474	0.0220*	
H7	0.42394	1.09075	0.12266	0.0244*	
H8	0.16011	1.07380	0.06663	0.0273*	
H9	0.12208	0.89666	−0.00482	0.0256*	
H11	0.689 (2)	0.667 (2)	0.0118 (8)	0.035 (5)*	
H11A	0.22112	0.64398	−0.04383	0.0365*	
H11B	0.42344	0.61199	−0.03771	0.0365*	
H11C	0.34041	0.75140	−0.07550	0.0365*	
H42	1.52737	0.82897	0.18763	0.0277*	
H43	1.68358	0.89864	0.27051	0.0318*	
H44	1.56251	0.86544	0.34963	0.0299*	
H45	1.28523	0.76294	0.34576	0.0282*	
H46	1.12358	0.70188	0.26270	0.0243*	

### Atomic displacement parameters (Å^2^)

**Table:**

	*U*^11^	*U*^22^	*U*^33^	*U*^12^	*U*^13^	*U*^23^
O1	0.0135 (2)	0.0198 (3)	0.0159 (3)	0.0031 (2)	−0.0012 (2)	−0.0034 (2)
O2	0.0225 (3)	0.0288 (4)	0.0201 (3)	0.0093 (3)	−0.0006 (3)	−0.0063 (3)
N11	0.0131 (3)	0.0170 (3)	0.0161 (3)	0.0010 (2)	−0.0014 (2)	−0.0017 (2)
N31	0.0271 (5)	0.0631 (8)	0.0330 (6)	0.0232 (5)	−0.0089 (4)	−0.0117 (5)
C2	0.0142 (3)	0.0206 (4)	0.0159 (3)	0.0032 (3)	0.0001 (3)	−0.0008 (3)
C3	0.0126 (3)	0.0228 (4)	0.0153 (3)	0.0029 (3)	−0.0011 (2)	−0.0010 (3)
C4	0.0117 (3)	0.0198 (3)	0.0145 (3)	−0.0008 (2)	0.0000 (2)	−0.0004 (3)
C4A	0.0113 (3)	0.0179 (3)	0.0155 (3)	−0.0007 (2)	−0.0001 (2)	−0.0016 (3)
C5	0.0137 (3)	0.0196 (4)	0.0190 (4)	−0.0004 (3)	−0.0005 (3)	−0.0045 (3)
C6	0.0143 (3)	0.0198 (4)	0.0202 (4)	0.0004 (3)	0.0004 (3)	−0.0051 (3)
C6A	0.0124 (3)	0.0171 (3)	0.0169 (3)	0.0003 (2)	0.0003 (2)	−0.0015 (3)
C6B	0.0125 (3)	0.0174 (3)	0.0177 (3)	0.0012 (3)	0.0006 (3)	−0.0002 (3)
C7	0.0156 (3)	0.0235 (4)	0.0215 (4)	0.0045 (3)	0.0012 (3)	−0.0015 (3)
C8	0.0152 (3)	0.0269 (4)	0.0254 (4)	0.0054 (3)	0.0002 (3)	0.0007 (3)
C9	0.0140 (3)	0.0257 (4)	0.0229 (4)	0.0022 (3)	−0.0022 (3)	0.0025 (3)
C10	0.0144 (3)	0.0199 (4)	0.0179 (4)	−0.0007 (3)	−0.0018 (3)	0.0017 (3)
C10A	0.0125 (3)	0.0167 (3)	0.0165 (3)	0.0004 (2)	−0.0002 (2)	0.0011 (3)
C11	0.0231 (4)	0.0252 (4)	0.0226 (4)	−0.0012 (3)	−0.0039 (3)	−0.0029 (3)
C11A	0.0117 (3)	0.0162 (3)	0.0149 (3)	0.0000 (2)	−0.0003 (2)	−0.0006 (3)
C11B	0.0121 (3)	0.0157 (3)	0.0151 (3)	0.0003 (2)	0.0006 (2)	−0.0013 (3)
C31	0.0183 (4)	0.0356 (5)	0.0200 (4)	0.0082 (3)	−0.0028 (3)	−0.0046 (4)
C41	0.0136 (3)	0.0217 (4)	0.0150 (3)	−0.0001 (3)	−0.0010 (3)	−0.0007 (3)
C42	0.0145 (3)	0.0359 (5)	0.0179 (4)	−0.0040 (3)	−0.0011 (3)	−0.0015 (3)
C43	0.0177 (4)	0.0372 (5)	0.0222 (4)	−0.0034 (3)	−0.0050 (3)	−0.0028 (4)
C44	0.0261 (4)	0.0281 (5)	0.0180 (4)	0.0025 (3)	−0.0055 (3)	−0.0032 (3)
C45	0.0292 (4)	0.0256 (4)	0.0150 (4)	0.0006 (3)	0.0013 (3)	−0.0006 (3)
C46	0.0201 (4)	0.0236 (4)	0.0170 (4)	−0.0016 (3)	0.0024 (3)	−0.0006 (3)

### Geometric parameters (Å, º)

**Table:**

O1—C2	1.3670 (11)	C10—C10A	1.4056 (13)
O1—C11B	1.3748 (11)	C10—C11	1.4967 (14)
O2—C2	1.2137 (12)	C11A—C11B	1.3956 (12)
N11—C10A	1.3906 (11)	C41—C46	1.4016 (14)
N11—C11A	1.3772 (12)	C41—C42	1.3952 (13)
N31—C31	1.1548 (17)	C42—C43	1.3929 (16)
N11—H11	0.874 (18)	C43—C44	1.3874 (16)
C2—C3	1.4565 (14)	C44—C45	1.3914 (16)
C3—C4	1.3748 (13)	C45—C46	1.3942 (14)
C3—C31	1.4312 (14)	C5—H5	0.9500
C4—C41	1.4845 (14)	C6—H6	0.9500
C4—C4A	1.4420 (12)	C7—H7	0.9500
C4A—C5	1.4230 (13)	C8—H8	0.9500
C4A—C11B	1.4020 (13)	C9—H9	0.9500
C5—C6	1.3767 (13)	C11—H11A	0.9800
C6—C6A	1.4074 (14)	C11—H11B	0.9800
C6A—C6B	1.4400 (12)	C11—H11C	0.9800
C6A—C11A	1.4120 (12)	C42—H42	0.9500
C6B—C10A	1.4139 (13)	C43—H43	0.9500
C6B—C7	1.4032 (13)	C44—H44	0.9500
C7—C8	1.3828 (14)	C45—H45	0.9500
C8—C9	1.4064 (14)	C46—H46	0.9500
C9—C10	1.3946 (13)		
			
C2—O1—C11B	121.34 (8)	O1—C11B—C4A	122.89 (7)
C10A—N11—C11A	107.95 (7)	C4A—C11B—C11A	119.92 (8)
C11A—N11—H11	126.4 (11)	N31—C31—C3	178.26 (13)
C10A—N11—H11	125.2 (11)	C4—C41—C42	120.22 (8)
O1—C2—C3	116.93 (8)	C42—C41—C46	119.29 (9)
O2—C2—C3	124.88 (9)	C4—C41—C46	120.47 (8)
O1—C2—O2	118.12 (9)	C41—C42—C43	120.64 (9)
C4—C3—C31	122.63 (9)	C42—C43—C44	119.91 (10)
C2—C3—C4	122.66 (8)	C43—C44—C45	119.90 (10)
C2—C3—C31	114.44 (8)	C44—C45—C46	120.52 (9)
C4A—C4—C41	120.97 (8)	C41—C46—C45	119.72 (9)
C3—C4—C4A	118.33 (9)	C4A—C5—H5	119.00
C3—C4—C41	120.69 (8)	C6—C5—H5	119.00
C4—C4A—C5	123.02 (9)	C5—C6—H6	120.00
C4—C4A—C11B	117.56 (8)	C6A—C6—H6	120.00
C5—C4A—C11B	119.39 (8)	C6B—C7—H7	121.00
C4A—C5—C6	121.03 (9)	C8—C7—H7	121.00
C5—C6—C6A	119.17 (8)	C7—C8—H8	120.00
C6B—C6A—C11A	106.06 (8)	C9—C8—H8	120.00
C6—C6A—C6B	133.32 (8)	C8—C9—H9	119.00
C6—C6A—C11A	120.59 (8)	C10—C9—H9	119.00
C6A—C6B—C10A	106.77 (7)	C10—C11—H11A	109.00
C6A—C6B—C7	132.76 (9)	C10—C11—H11B	109.00
C7—C6B—C10A	120.45 (8)	C10—C11—H11C	109.00
C6B—C7—C8	118.20 (9)	H11A—C11—H11B	109.00
C7—C8—C9	120.61 (9)	H11A—C11—H11C	109.00
C8—C9—C10	122.95 (9)	H11B—C11—H11C	109.00
C9—C10—C11	121.45 (9)	C41—C42—H42	120.00
C10A—C10—C11	122.73 (8)	C43—C42—H42	120.00
C9—C10—C10A	115.82 (9)	C42—C43—H43	120.00
C6B—C10A—C10	121.97 (8)	C44—C43—H43	120.00
N11—C10A—C6B	109.13 (7)	C43—C44—H44	120.00
N11—C10A—C10	128.88 (8)	C45—C44—H44	120.00
C6A—C11A—C11B	119.77 (9)	C44—C45—H45	120.00
N11—C11A—C11B	130.08 (8)	C46—C45—H45	120.00
N11—C11A—C6A	110.08 (7)	C41—C46—H46	120.00
O1—C11B—C11A	117.18 (8)	C45—C46—H46	120.00
			
C11B—O1—C2—O2	−179.96 (9)	C6—C6A—C6B—C10A	−177.82 (10)
C11B—O1—C2—C3	3.08 (12)	C11A—C6A—C6B—C7	178.07 (10)
C2—O1—C11B—C4A	−3.74 (13)	C11A—C6A—C6B—C10A	−0.10 (10)
C2—O1—C11B—C11A	177.50 (8)	C6—C6A—C11A—N11	178.70 (8)
C11A—N11—C10A—C6B	0.83 (10)	C6—C6A—C11A—C11B	1.36 (13)
C11A—N11—C10A—C10	−177.20 (9)	C6B—C6A—C11A—N11	0.62 (10)
C10A—N11—C11A—C6A	−0.91 (10)	C6B—C6A—C11A—C11B	−176.72 (8)
C10A—N11—C11A—C11B	176.08 (9)	C6A—C6B—C7—C8	−177.19 (10)
O1—C2—C3—C4	1.63 (14)	C10A—C6B—C7—C8	0.78 (14)
O1—C2—C3—C31	175.76 (8)	C6A—C6B—C10A—N11	−0.45 (10)
O2—C2—C3—C4	−175.11 (10)	C6A—C6B—C10A—C10	177.75 (8)
O2—C2—C3—C31	−0.98 (14)	C7—C6B—C10A—N11	−178.89 (8)
C2—C3—C4—C4A	−5.55 (14)	C7—C6B—C10A—C10	−0.70 (14)
C2—C3—C4—C41	173.15 (9)	C6B—C7—C8—C9	−0.04 (15)
C31—C3—C4—C4A	−179.20 (9)	C7—C8—C9—C10	−0.85 (16)
C31—C3—C4—C41	−0.50 (14)	C8—C9—C10—C10A	0.92 (14)
C3—C4—C4A—C5	−173.02 (9)	C8—C9—C10—C11	−179.30 (10)
C3—C4—C4A—C11B	4.82 (13)	C9—C10—C10A—N11	177.66 (9)
C41—C4—C4A—C5	8.28 (14)	C9—C10—C10A—C6B	−0.15 (13)
C41—C4—C4A—C11B	−173.88 (8)	C11—C10—C10A—N11	−2.12 (15)
C3—C4—C41—C42	54.88 (13)	C11—C10—C10A—C6B	−179.93 (9)
C3—C4—C41—C46	−123.43 (10)	N11—C11A—C11B—O1	4.06 (14)
C4A—C4—C41—C42	−126.45 (10)	N11—C11A—C11B—C4A	−174.74 (9)
C4A—C4—C41—C46	55.24 (13)	C6A—C11A—C11B—O1	−179.21 (8)
C4—C4A—C5—C6	179.91 (9)	C6A—C11A—C11B—C4A	2.00 (13)
C11B—C4A—C5—C6	2.10 (14)	C4—C41—C42—C43	−179.49 (10)
C4—C4A—C11B—O1	−0.34 (13)	C46—C41—C42—C43	−1.16 (16)
C4—C4A—C11B—C11A	178.38 (8)	C4—C41—C46—C45	178.19 (9)
C5—C4A—C11B—O1	177.59 (8)	C42—C41—C46—C45	−0.14 (15)
C5—C4A—C11B—C11A	−3.69 (13)	C41—C42—C43—C44	1.27 (18)
C4A—C5—C6—C6A	1.22 (14)	C42—C43—C44—C45	−0.06 (19)
C5—C6—C6A—C6B	174.51 (10)	C43—C44—C45—C46	−1.25 (17)
C5—C6—C6A—C11A	−2.95 (14)	C44—C45—C46—C41	1.34 (16)
C6—C6A—C6B—C7	0.35 (18)		

### Hydrogen-bond geometry (Å, º)

**Table:**

*D*—H···*A*	*D*—H	H···*A*	*D*···*A*	*D*—H···*A*
N11—H11···O2^i^	0.874 (18)	2.095 (19)	2.9561 (11)	168.2 (15)
C43—H43···N31^ii^	0.95	2.56	3.3130 (17)	136

Symmetry codes: (i) −*x*+2, −*y*+1, −*z*; (ii) −*x*+7/2, *y*+1/2, −*z*+1/2.
